# Plant resistance against the parasitic nematode *Heterodera schachtii* is mediated by MPK3 and MPK6 kinases, which are controlled by the MAPK phosphatase AP2C1 in Arabidopsis

**DOI:** 10.1093/jxb/erv440

**Published:** 2015-10-05

**Authors:** Ekaterina Sidonskaya, Alois Schweighofer, Volodymyr Shubchynskyy, Nina Kammerhofer, Julia Hofmann, Krzysztof Wieczorek, Irute Meskiene

**Affiliations:** ^1^Division of Plant Protection, Department of Crop Sciences, University of Natural Resources and Life Sciences, Konrad-Lorenz-Straße 24, A-3430 Tulln on the Danube, Austria; ^2^Max F. Perutz Laboratories of the University and Medical University of Vienna, Dr Bohr-Gasse 9, A-1030 Vienna, Austria; ^3^Institute of Biotechnology, University of Vilnius, Graiciuno 8, LT-02242 Vilnius, Lithuania; ^4^Department of Ecogenomics and Systems Biology, University of Vienna, Althanstraße 14, A-1090 Vienna, Austria

**Keywords:** *Heterodera schachtii*, MAPK phosphatase, MAPK signalling, PP2C phosphatase, plant-pathogen response, plant–nematode interactions.

## Abstract

Plant-parasitic cyst nematodes infect plants and form highly sophisticated feeding sites in roots. It is not known which plant cell signalling mechanisms trigger plant defence during the early stages of nematode parasitism. Mitogen-activated protein kinases (MAPKs) are central components of protein phosphorylation cascades transducing extracellular signals to plant defence responses. MAPK phosphatases control kinase activities and the signalling outcome. The involvement and the role of MPK3 and MPK6, as well as the MAPK phosphatase AP2C1, is demonstrated during parasitism of the beet cyst nematode *Heterodera schachtii* in *Arabidopsis*. Our data reveal notable activation patterns of plant MAPKs and the induction of AP2C1 suggesting the attenuation of defence signalling in plant cells during early nematode infection. It is demonstrated that the *ap2c1* mutant that is lacking AP2C1 is more attractive but less susceptible to nematodes compared with the AP2C1-overexpressing line. This implies that the function of AP2C1 is a negative regulator of nematode-induced defence. By contrast, the enhanced susceptibility of *mpk3* and *mpk6* plants indicates a positive role of stress-activated MAPKs in plant immunity against nematodes. Evidence is provided that phosphatase AP2C1, as well as AP2C1-targeted MPK3 and MPK6, are important regulators of plant–nematode interaction, where the co-ordinated action of these signalling components ensures the timely activation of plant defence.

## Highlight

In plant immunity against nematodes, MPK3/6 act positively and their activity is controlled by the MAPK phosphatase AP2C1. The MAPK activation pattern suggests the attenuation of defence signalling during nematode infection.

## Introduction

Sedentary plant-parasitic nematodes, cyst, and root-knot nematodes are the most successful microscopic root endoparasites (Burrows, 1992). The sugar beet cyst nematode *Heterodera schachtii* is specialized to exploit Brassicaceae and Chenopodiaceae species mainly, including the model plant *Arabidopsis thaliana* (Sijmons *et al.*, 1991). During the infection process, the nematode second stage juvenile (J2) penetrates the epidermis along the entire root with destructive stylet thrusting and facilitates this by secreting numerous cell wall digesting enzymes that are produced in subventral glands (Wyss, 1992; Goellner *et al.*, 2001). Inside the roots, J2 migrates intracellularly towards the vasculature, severely damaging root cells and resulting in a path of necrotic cells (Hussey and Grundler, 1998). Within the central cylinder, J2 selects a single parenchymatous cell which serves as the initial syncytial cell (ISC) (Wyss and Zunke, 1986; Wyss, 1992; Wyss and Grundler, 1992). With the stylet, the nematode punctures the cell wall, whereas the plasma membrane remains carefully invaginated. The stylet stays protruded for several hours during which the J2 does not feed. A few hours afterwards, the juvenile starts to withdraw solutes and an increase in cytoplasmic streaming, the proliferation of organelles, alterations in cell wall architecture, and an enlargement of the nuclei (Wyss, 1992) are the first changes in the ISC. Now the host provides all the nutrients necessary for the completion of the nematode’s life cycle as well as the development and maintenance of the feeding site.

During the two weeks after infection, larvae moult three times to develop into either an adult female or male. After fertilization, the dead body of the female forms a cyst that serves as the outlasting stage. The nematode’s behaviour during the infection process and its subsequent development has been intensively studied; however, the early responses of host plant cells are poorly understood. During the nematode’s root penetration and intracellular migration the host may perceive the mechanical damage of the cell wall or deformation of the plasma membrane (Wieczorek and Seifert, 2012) to induce versatile cell signalling cascades. Generally, host cells recognize pathogen-associated molecular patterns (PAMPs) or damage-associated molecular patterns (DAMPs). Plant-parasitic nematodes could derive such molecules from the surface of the J2s or secrete actively from amphids or gland cells (Vieira *et al.*, 2011). It has been confirmed that, during migration, the J2s release widely unknown effectors from their subventral glands into the apoplast (Vieira *et al.*, 2011; Eves-van den Akker *et al.*, 2014) that may interact with plant cell receptors and signalling cascades (Vieira *et al.*, 2011; Jaouannet *et al.*, 2012). It is also suggested that nematode secretions produced in the dorsal gland during the sedentary stage are responsible for the reorganization of the affected ISCs. They are injected into the syncytium’s cytoplasm and are necessary for its establishment and maintenance. It is currently speculated that these secretions are able to modulate plant signalling involved in the recognition of the nematode by the plant leading to the reduction or deactivation of plant defence responses (Hewezi and Baum, 2013). Several effectors, such as cellulose-binding protein (Ding *et al.*, 1998; Hewezi *et al.*, 2008), Hs19C07, from *H. schachtii* (Lee *et al.*, 2011) or effectors that target defence- and stress-associated proteins (Hewezi *et al.*, 2010; Patel *et al.*, 2010; Hamamouch *et al.*, 2012; Hewezi and Baum, 2013) were recently characterized. Nematode effectors might be recognized by plant plasma membrane-bound receptor-like kinases (RLKs) which, in turn, would lead to the activation of mitogen-activated protein kinases (MAPKs) that are central components of cell signalling cascades (Cazale *et al.*, 1999; Kiegerl *et al.*, 2000; Asai *et al.*, 2002; Doczi *et al.*, 2007). MAPKs are active when the conserved TEY motif at the activation loop of the kinase is phosphorylated by a dual specificity kinase (MAPKK) on both Thr and Tyr (Kiegerl *et al.*, 2000). Activated MAPKs mediate multiple plant defence responses (Meng and Zhang, 2013). In *Arabidopsis*, the activation of MPK3 and MPK6 in response to PAMPs and DAMPs and their roles in plant–pathogen interactions has been demonstrated (Han *et al.*, 2010; Galletti *et al.*, 2011; Tena *et al.*, 2011; Meng and Zhang, 2013). MPK3 and MPK6 are positive regulators of plant defence responses controlling ethylene (ET) (Tena *et al.*, 2011; Meng and Zhang, 2013) and jasmonate (JA) biosynthesis (Schweighofer and Meskiene, 2008). MPK3 and MPK6, together with JA, are essential for plant defence against *Botrytis cinerea* as *mpk3* (Ren *et al.*, 2008) and *mpk6* (Mendez-Bravo *et al.*, 2011) mutants, as well as lines with attenuated MAPK activities (Schweighofer *et al.*, 2007) were impaired in defence against this necrotroph. Beside the contribution by individual MAPK proteins, cellular stress responses could be influenced by the intensity and duration of the MAPK activation determined by the dephosphorylation of the kinase executed by the MAPK phosphatases (Caunt and Keyse, 2013). Dephosphorylation and inactivation of plant stress-activated MAPKs can be performed by dual specificity phosphatases (DSP) (Bartels *et al.*, 2010) as well as PP2C-type Ser/Thr phosphatases (Meskiene *et al.*, 1998, 2003; Meskiene and Hirt, 2000; Umbrasaite *et al.*, 2010; Fuchs *et al.*, 2013). The *Arabidopsis* PP2C-type MAPK phosphatases play an important role in the regulation of wound- or pathogen-related signal transduction and activation of plant defence responses (Schweighofer *et al.*, 2007; Umbrasaite *et al.*, 2010; Galletti *et al.*, 2011; Fuchs *et al.*, 2013). AP2C1 negatively regulates wound-induced MAPK activities in *Arabidopsis* leaves and AP2C1-overexpressing plants produce less wound-induced ET and are more susceptible to *B. cinerea* than the wild type (Schweighofer *et al.*, 2007). On the other hand, the *ap2c1* mutant exhibits higher JA levels in response to wounding and is more resistant to the phytophagous mite *Tetranychus urticae* (Schweighofer *et al.*, 2007). In contrast to more advanced knowledge of cell signalling during microbe– or herbivore–plant interaction the involvement of MAPKs and their phosphatases during plant–nematode interaction has not been studied so far. The only evidence is reported on the expression of a tomato MAPK gene, a homologue of *Arabidopsis* MPK9, induced 25 d post infection (dpi) with the root-knot nematode *Meloidogyne javanica* (Wang *et al.*, 2003). This gap in knowledge provides notable questions about MAPK signalling activation triggered by plant-parasitic nematodes and its connection to plant defence against these parasites.

This is a study on the signalling processes during the early stages of nematode parasitism. The role of the signalling pathway was investigated via MPK3 and MPK6 and their negative regulator, the PP2C-type MAPK phosphatase AP2C1, during the interaction between *Arabidopsis* and the cyst nematode *H. schachtii*. *AP2C1* expression was analysed during different stages of early *H. schachtii* infection and syncytium formation compared with mechanically wounded roots. Different activation patterns of MPK3 and MPK6 were demonstrated in the wild type and the *ap2c1* line that correlate with the enhanced resistance of the *ap2c1* mutant to nematodes compared with the AP2C1-overexpressing line. These results indicate an important role of AP2C1 as a negative regulator and a positive role of the AP2C1-regulated kinases MPK3 and MPK6 in plant resistance against *H. schachtii*.

## Materials and methods

### Plant material and growth conditions

Following *Arabidopsis thaliana* lines in the Col-0 genetic background were used: pAP2C1::GUS line, *ap2c1,* AP2C1-oe, AP2C1-comp (Schweighofer *et al.*, 2007)*, mpk6-2* (SALK_127507), and *mpk3-1* (SALK_151594)*. A. thaliana* Col-0 was used as the wild type (WT). The pAP2C1::AP2C1-GFP line was generated by replacing the CaMV 35S promoter of pGreenII0029-35S-AP2C1-GFP vector (Schweighofer *et al.*, 2007) with the 1.3kb AP2C1 promoter region (Schweighofer *et al.*, 2007) generating the pGreenII0029-pAP2C1::AP2C1-GFP construct. *Agrobacteria* strain GV3101-pMP90-pSoup was transformed by electroporation and positive clones checked by PCR. Col-0 plants were transformed by the floral dip method and positive plants were selected on MS plates containing kanamycin and by fluorescence microscopy. Plants were cultured *in vitro* under sterile conditions on Knop medium at 21 °C under a 16/8h photoperiod (Sijmons *et al.*, 1991). For qRT-PCR, GUS, Western blot measuring kinase activity upon nematode infection, wounding experiments, and the estimation of infection rate in the WT and *ap2c1* lines, plants were grown on glass discs (approximately 7cm in diameter) with a thin layer of Knop medium (2–3mm). This procedure was applied to ensure rapid nematode infection and to provide good settings for monitoring the infection process under the microscope. For the infection tests, plants were grown in Petri dishes (94mm in diameter) containing a thick layer of Knop medium (5–7mm) with 10 plants per plate. Prior to the kinase assay and Western blot, seedlings were grown for 16 d in 4.5ml of 1/2 strength MS liquid medium. For wounding experiments (qRT-PCR, and Western blot) 10-d-old roots were wounded by gently squeezing with forceps and subsequently kept prior to RNA or protein extraction in a growth chamber.

### Nematode infection assays

Before inoculation, the total root length of the seedlings was estimated (Bohlmann and Wieczorek, 2015). For qRT-PCR, Western blot analysis, wounding experiments, and GUS analysis, 10-d-old plants were inoculated with approximately 100 J2s per plant. A high number of J2s was applied to boost the plant responses. For nematode infection tests the method described in Bohlmann and Wieczorek (2015) was used. Ten-day-old plants were inoculated with 50 freshly hatched J2s and, after 14 d, the total number of female nematodes was counted. Results were obtained from three biological replicates including approximately 50 plants per replicate and line. For an estimation of the infection rate in 3 or 4 plants per line per plate were inoculated with 50 J2s. For each of three biological replicates, four plates were used. For this analysis, as well as GUS and Western blot, migrating J2s were monitored microscopically to estimate the time point of syncytium initiation [0 hours after syncytium initiation (hasi)], which is manifested as the cessation of both nematode movement and stylet thrusting (according to Wyss, 1992). Statistical differences were calculated using STATGRAPHICS plus 5.0 software with the one-way ANOVA test.

### RNA extraction, cDNA synthesis, and qRT-PCR

Whole roots, except root tips, were collected at 1min, 0.5, 2.5, 30, and 48h after inoculation (hai) and frozen in liquid nitrogen. RNA was extracted using the RNeasy Plant Mini Kit (Qiagen, Germany) according to the manufacturer’s instructions. Non-infected roots, excluding root tips, were used as a control. The amount and quality of the RNA was controlled using an Agilent 2100 bioanalyser (Agilent Technologies, USA). Reverse transcription was performed with the SuperScript III kit (Invitrogen, Carlsbad, CA, USA) using random primers. The relative change in expression levels of synthesized transcripts was measured using the 7300 Real Time PCR System (Applied Biosystems, USA). *18S rRNA* and *UBP22* were used as the endogenous controls (Hofmann and Grundler, 2007). Primers for qRT-PCR were designed using the primer-BLAST (http://www.ncbi.nlm.nih.gov/tools/primer-blast/). The following *AP2C1* primers were used: forward 5′-ACTTGGCAACAGACGCGTCGT-3′, reverse 5′- GCACGACAGTCACCGGCGTT-3′. The efficiencies of primers were tested by standard quantification, using standard curves of four template dilutions and based on three separate replicates. Changes in expression were calculated according to the 2^–∆∆Ct^ method (Livak and Schmittgen, 2001).

### Histochemical GUS assay

For histochemical detection of β-glucuronidase activity (Jefferson *et al.*, 1987) plants were grown and treated as described above. The stage of nematode infection was determined (Siddique *et al.*, 2014). At 4, 15, 24, 30, and 48 hasi the root regions containing nematodes were excised and fixed in 0.5% glutaraldehyde for 15min at room temperature (RT) and then washed in distilled water. Subsequently, the seedlings were incubated for 12–18h at 37 °C in staining solution containing 1mM 5-bromo-4-chloro-3-indolyl-β-D-glucuronic acid (X-Glc), 0.05M sodium phosphate (pH 7.0), 0.5mM K_4_[Fe(CN)_6_], 0.5mM K_3_[Fe(CN)_6_], and 0.05% Triton X-100. After the staining procedure, samples were cleared using lactic acid for 24–48h and staining was analysed under the light microscope (Zeiss Axiovert 200M, Germany).

### Confocal microscopy

Microscopic localization of GFP fusion protein in *Arabidopsis* pAP2C1::AP2C1-GFP roots and nematode-feeding sites was performed using confocal laser scanning microscope (TCS SP 5; Leica Microsystems). The images were taken under a ×63 water objective. Confocal images were processed using Leica Confocal Software and PHOTOSHOP CS3.

### Kinase assays

Prior to the kinase assay, 0.1mg ml^–1^ cellulase was added to the 1/2 strength MS liquid media containing 16-d-old seedlings. Subsequently, roots were incubated for 10min and 30min, collected, and immediately frozen in liquid nitrogen prior to protein extraction. Immunoprecipitation of MAPKs was performed according to Schweighofer *et al.* (2009). Briefly, samples were incubated in 15 μl of kinase buffer containing 1.5 μg myelin basic protein (MBP) and 3 μCi [γ-^32^P] ATP for 30min at RT. Adding 4× SDS-PAGE sample buffer stopped the reaction. Samples were heated at 95 °C for 3min and run on a 12.5% SDS-PAA gel. Gels were then stained in Coomassie Blue staining solution [0.25% (w/v) Coomassie Brilliant Blue R-250, 45% (v/v) methanol, 10% (v/v) acetic acid] for 5min and distained with regular changes of distaining solution [45% (v/v) methanol, 10% (v/v) acetic acid] for 1.5h. Subsequently, the gels were placed on 3mm Whatman paper and dried at 80 °C for 1h. The phosphorylation of MBP was analysed by autoradiography.

### Protein extraction and purification

Whole roots after mechanical wounding, except root tips, were collected at 0, 1, 10, 30, 60, and 210min after treatment and immediately frozen in liquid nitrogen for further protein extraction. Whole nematode-infected roots, except root tips, were collected at 0, 0.5, 2.5, 6, and 20 hai and immediately frozen in liquid nitrogen for further protein extraction. 50–70mg root tissue was disrupted by grinding in liquid nitrogen in 100–200 μl Lacus buffer, containing 25mM Tris-HCl, pH 7.8, 10mM MgCl_2_, 15mM EGTA, 75mM NaCl, 1mM dithiothreitol, 1mM NaF, 0.5mM NaVO_3_, 15mM β-glycerophosphate, 0.1% (v/v) Tween-20, 15mM *p*-nitrophenylphosphate, 0.5mM phenylmethylsulphonylfluoride (PMSF), leupeptine (5mg ml^–1^), aprotinin (5mg ml^–1^). The homogenate was centrifuged for 35min at 16 000rpm at 4 °C. Protein concentration was determined by the Bradford assay. For immunoprecipitation, 100 μg of total protein extract was incubated on a rotating shaker with 20 μl of protein A sepharose beads (Amersham Biosciences) together with 2 μl anti MPK6 antibody (Sigma) for 12–16h at 4 °C. Afterwards, beads were washed three times with washing buffer (50mM Tris, pH 7.4, 250mM NaCl, 5mM EGTA, 5mM EDTA, 0.1% (v/v) Tween-20, 5mM NaF, 0.1% (v/v) Nonidet P-40, 0.5mM PMSF).

### Western blot

Western blot analysis was performed according to Meskiene *et al.* (2003) except for the analysis determining the protein phosphorylation status. For this purpose, extracted proteins were separated on 12.5% SDS-PAA gel and transferred to a nitrocellulose membrane, using Biorad Trans-Blot SD Semi-Dry Transfer Cell at 15–20V for 20min. The membrane was probed with the following antibody concentrations: phospho-p44/42 MAPK (Erk1/2) (Thr202/Tyr204) rabbit mAb, 1:2 000 (Cell Signalling Technology, http://www.cellsignal.com) as primary antibody and alkaline phosphatase anti-rabbit IgG (H+L), 1:1 000 (Vector Laboratories), as secondary antibody. MPK6 was detected with an MPK6-specific antibody (1:5 000) from Sigma. Each Western blot experiment was repeated at least three times covering two independent biological replicates.

### Attraction assay

The nematode attraction assay was performed according to Dalzell *et al.* (2011) and Kammerhofer *et al.* (2015). Ten-day-old seedlings were grown and inoculated as described above. Plates containing 2% water agar with cylindrical counting wells (8mm in diameter) connected via a cylindrical channel (20×2.5mm) were prepared. Agar discs containing root exudates either from non-treated or treated *ap2c1* or wild-type plants were obtained 2 d after treatment and placed into the counting wells. One hundred J2s were placed in the middle of the connecting channel. After 20h, sodium azide (1 μl 1mM) was applied (McMiller and Johnson, 2005) in order to arrest the nematodes on the spot. Experiments were performed with three independent replicates with at least three plates per line.

### Accession numbers

Sequence data from this article can be found in the *Arabidopsis* Genome Initiative or GenBank/EMBL databases under the following accession numbers: *MPK3* (At3g45640), *MPK6* (At2g43790), and *AP2C1* (At2g30020).

## Results

### MAPK activation during different phases of nematode infection, in response to wounding and cellulase treatment

**Fig. 1.**
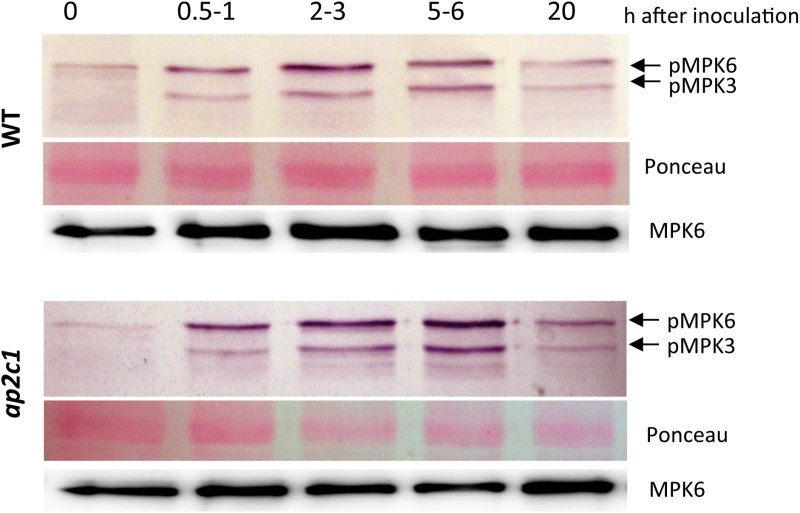
Analysis of MAPKs activation in roots during the early stage of *H. schachtii* infection. Phosphorylation of MPK6 and MPK3 was detected by immunoblotting with the anti-phospho ERK1/2 antibody. MPK6 protein amounts were detected with an MPK6-specific antibody. Ponceau-stained membranes present protein loading.

In *Arabidopsis* leaves, MAPKs are rapidly activated by biotic stresses (Asai *et al.*, 2002; Zipfel *et al.*, 2006; Suarez-Rodriguez *et al.*, 2007; Wu *et al.*, 2007) as well as by mechanical damage (Schweighofer *et al.*, 2007); however, the activation of MAPK signalling in roots after nematode infection has not been studied so far. Therefore, we were interested to discover whether MAPKs are activated during parasitism of *H. schachtii* in roots. Nematode-infected *A. thaliana* Col-0 root samples collected at different time points after inoculation (indicated as hours after inoculation, hai) were analysed for MAPK phosphorylation (Fig. 1; see Supplementary Fig. S3 at *JXB* online). An anti-phospho ERK1/2 antibody was used detecting phosphorylated (on the essential pTEpY motif of the MAPK) and thus activated MAPKs in plants. During nematode parasitism, the first activation of MPK6 and MPK3 was observed at 0.5 hai (Fig. 1). The maximum of MPK6 activation has been detected at 2 hai and remained at a high level until 6 hai. The highest activation of MPK3 was detected at 6 hai. At 20 hai, phosphorylation of MPK6 and MPK3 was reduced (Fig. 1; see Supplementary Fig. S3 at *JXB* online). The detection of MPK6 protein indicated unchanged protein amounts.

Since fast and severe damage is caused by the nematode entering and moving within the roots, the activation of MAPKs in mechanically damaged roots was tested (see Supplementary Fig. S1 at *JXB* online). Our data demonstrate low activities of MPK3 and MPK6 in roots at the beginning of the treatment, rapid induction by wounding (see Supplementary Fig. S1 at *JXB* online) at 1min with the maximum at 10min. At 30min and 60min after wounding, MAPK phosphorylation was strongly reduced and, at 210min, the signal intensity returned to the background level (see Supplementary Fig. S1 at *JXB* online). To validate the specificity of MPK3 and MPK6 phosphorylation in the wild-type Col-0 plants, *mpk3* and *mpk6* knockout mutant plants were used (see Supplementary Fig. S2 at *JXB* online). The identity of MPK6 and MPK3 phospho-proteins detected in roots of wild-type plants (see Supplementary Fig. S1 at *JXB* online 1) was confirmed by data indicating wound-induced MPK6 phosphorylation in *mpk3* and MPK3 phosphorylation in *mpk6* plants (see Supplementary Fig. S2 at *JXB* online). Taken together, these results show the rapid and transient dual phosphorylation of MPK3 and MPK6 in response to mechanical root injury.

**Fig. 2.**
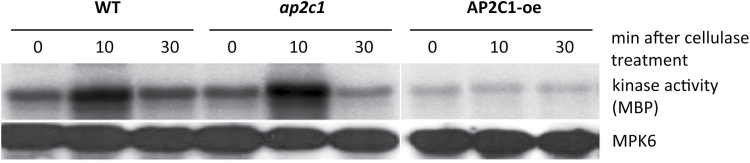
Analysis of MAPK activities in response to cellulase treatment. *Ex vivo* MPK6 activity from roots of Col-0, *ap2c1*, and AP2C1-oe lines at 0, 10, and 30min after cellulase treatment. Myelin basic protein (MBP) was used as a MAPK substrate; MPK6 protein amounts were detected with an MPK6-specific antibody.

During the infection process, the nematode injects saliva, which contains cellulase, into the root tissue (Wang *et al.*, 1999). Therefore it was tested if cellulase (from commercial stock) may activate MAPKs in roots (Fig. 2). The activities of MPK6, immunoprecipitated from the roots of wild-type plants, were assayed *ex vivo* by the MPK6 ability to phosphorylate myelin basic protein (MBP). Our results show that MPK6 was already strongly activated at 10min after treatment, whereas this activation had abated after 30min (Fig. 2). During cellulase-induced kinase activation, the protein amounts of MPK6 remained unchanged, suggesting that cellulase induces posttranslational modifications of the kinase rather than *de novo* production of the protein.

Taken together, these data demonstrate that MPK3 and MPK6 are activated during stress induced by nematode migration and syncytium formation as well as by mechanical damage and cellulase treatment. However, the profiles of nematode-induced kinase activity are clearly different from the patterns induced by mechanical damage or cellulase-treatment. In all cases, the transient activities of MAPKs suggest the action of protein phosphatases.

### Modulation of MAPK activation by the MAPK phosphatase AP2C1 during nematode migration and syncytium induction

To identify a potential regulator of MAPK signalling induced by nematodes, AP2C1 was selected due to its function as a wound-induced MAPK phosphatase (Schweighofer *et al.*, 2007). To evaluate the impact of AP2C1 on the regulation of MAPKs activities during nematode infection, the phosphorylation of MAPKs in response to nematode infection was studied in *ap2c1* knockout mutant plants which lack *AP2C1* due to T-DNA insertion (Schweighofer *et al.*, 2007). Figure 1, and Supplementary Fig. S3 at *JXB* online, show that the phosphorylation of MPK6 and MPK3 detected in *ap2c1* plants was stronger compared with the wild type.

It was also tested how modulation of *AP2C1* expression may affect the plant stress response induced by cell damage caused by cellulase by comparing MAPK activation in cellulase-treated roots of *ap2c1* and plants constitutively overexpressing *AP2C1* (AP2C1-oe) with the wild type. In *ex vivo* assays, MPK6 (Fig. 2) demonstrated higher kinase activities in *ap2c1* plants 10min after treatment than in the wild type, whereas no MPK6 activation was detected in the AP2C1-oe line (Fig. 2). Compared with the wild-type, MPK6 activity in AP2C1-oe was much lower at 0min as well as at 30min after cellulase application, suggesting the action of this phosphatase. The protein samples studied contained similar MPK6 protein amounts, indicating that modulations of kinase activities resulted from kinase activation/inactivation rather than from changes in protein accumulation (Fig. 2). These data indicate that MAPK activation is induced during nematode parasitism as well as triggered by cellulase, and suggest that AP2C1 may control MAPK signalling responses during these processes.

### Analysis of *AP2C1* expression upon *H. schachtii* infection

**Fig. 3.**
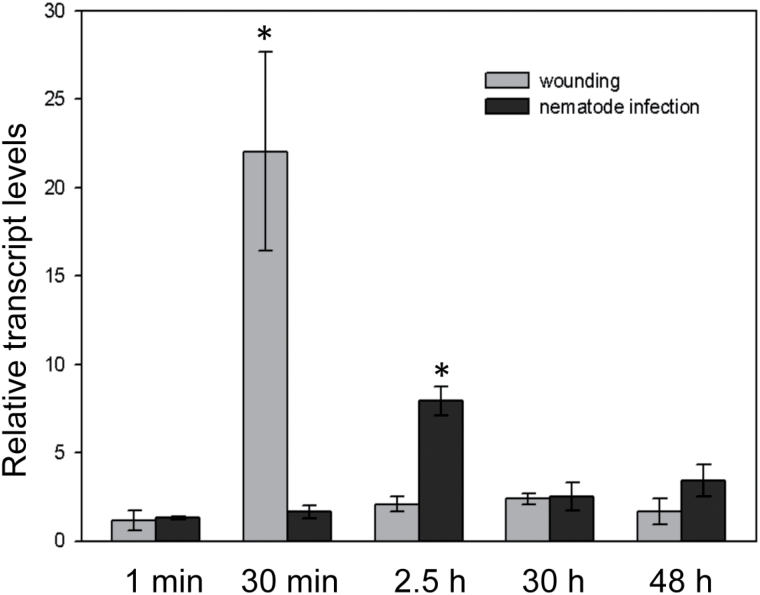
*AP2C1* expression in roots of wild-type *Arabidopsis* plants (Col-0). Changes in *AP2C1* expression in roots of Col-0 during the early stages of *H. schachtii* parasitism versus mechanical wounding compared with non-infected control plants. Relative expression levels were measured by qRT-PCR. Values are means ±SE (*n*=3). Asterisks represent significant differences: *P* < 0.05 versus control by ANOVA.

To determine whether *AP2C1* is involved in the host plant response triggered by parasitism of *H. schachtii*, the expression of this phosphatase was studied at different developmental stages of syncytial tissue by quantitative real-time PCR (qRT-PCR). Figure 1 demonstrates that the activation profile of MAPKs in response to nematode parasitism differs from the responses induced by wounding (see Supplementary Fig. S1 at *JXB* online). Therefore, the expression of *AP2C1* in root samples collected 1min, 0.5, 2.5, 30, and 48 hai or after mechanical wounding was quantified and compared (Fig. 3). Our data show that immediately after nematode inoculation or mechanical wounding (1min samples) there was no difference in the expression of *AP2C1* during both treatments compared with the non-treated control plants (Fig. 3). *AP2C1* expression was stable during J2s penetration, whereas at 2.5 hai an 8-fold increase of expression was observed (Fig. 3). This time point in our experiment corresponds to the phase when the first nematodes migrate within the root tissue and search for the ISC to induce the feeding sites. By contrast, a strong (~22-fold) induction of *AP2C1* expression was observed at 0.5h after wounding that decreased to the control level 2.5h later (Fig. 3). Thus, our data show a very fast and strong reaction in plant roots 30min after mechanical wounding manifested as highly enhanced expression of *AP2C1*. Interestingly, *AP2C1* is not induced at the same time during nematode infection but its first comparably low activation was observed later.

**Fig. 4.**
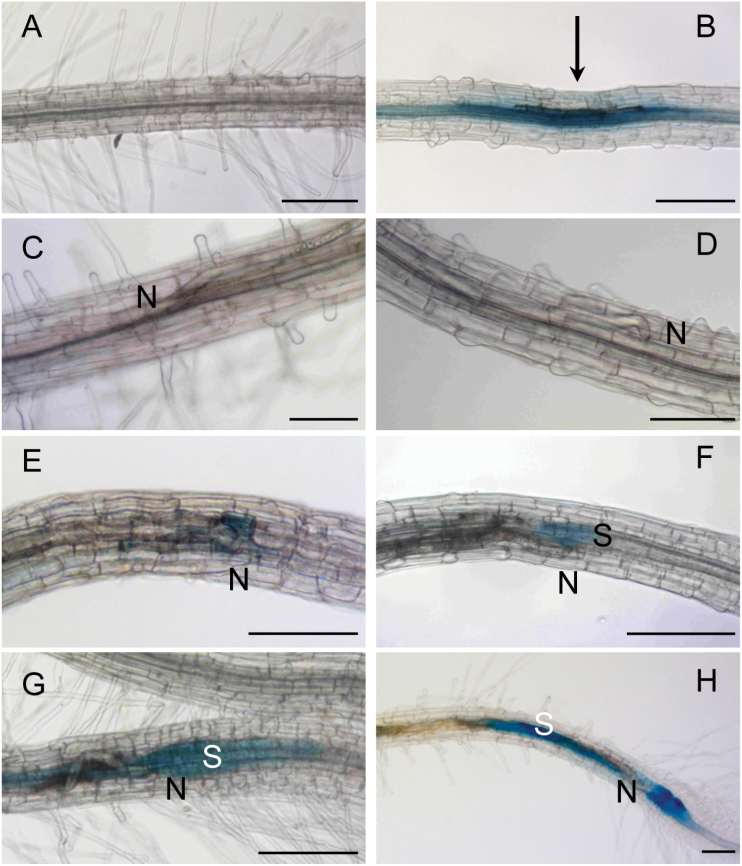
Microscopic analysis of *AP2C1* promoter activity during *H. schachtii* infection in roots. *AP2C1* promoter activity in (A) untreated pAP2C1::GUS root, (B) 1h after mechanical wounding (arrow points at the place of needle application), (C) during migration stage 2h after nematode infection (hai), (D) 4h after syncytium induction (hasi), (E) 15 hasi, (F) 24 hasi, (G) 30 hasi, and (H) 48 hasi. S, syncytium, N, head of the nematode. Bars: 200 μm.

In order to study where in the root *AP2C1* expression is induced in response to nematode parasitism, pAP2C1::GUS plants were analysed, where the expression of the β-glucuronidase (GUS) reporter gene was driven by the *AP2C1* promoter region (Schweighofer *et al.*, 2007). Our data demonstrate that, in non-infected roots, slight GUS staining was detectable in the central cylinder (Fig. 4A), whereas strong activation of pAP2C1 was detected 1h after mechanical wounding at the affected area (Fig. 4B). A time-course study was performed in roots during nematode migration 2h after infection (2 hai) and 4, 15, 24, 30, and 48 hours after syncytium induction (hasi) (Fig. 4). *AP2C1* promoter activity during migration (2 hai) was not detected (Fig. 4C) and during the first hours of syncytium induction (4 hasi, Fig. 4D). GUS activity was detected in syncytia at 15 hasi (Fig. 4E) and it was much increased at 24–48 hasi (Fig. 4F–H).

**Fig. 5.**
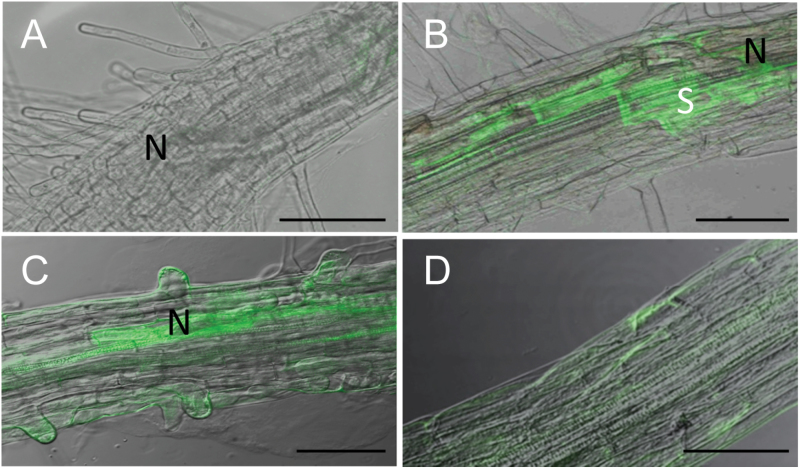
Microscopic analysis of AP2C1-GFP protein accumulation after nematode infection in roots. (A) Confocal image of wild-type root after *H. schachtii* infection at 15 hai. (B) Localization of AP2C1-GFP in *Arabidopsis* roots at 15 hai. (C) Localization of AP2C1-GFP in *Arabidopsis* roots at 24 hai. (D) Control: uninfected pAP2C1::AP2C1-GFP root. S, syncytium, N, head of the nematode. Bars: (A, B, C) 100 μm; (D) 200 μm.

In order to investigate if the activation of the *AP2C1* promoter in affected tissues leads to protein production, the localization of AP2C1 protein was analysed in its native domain by studying the expression of AP2C1-GFP fusion protein regulated by its own promoter in pAP2C1::AP2C1-GFP plants during nematode infection. Our results demonstrate no GFP fluorescence in control wild-type roots, however, weak autofluorescence originating from necrotic cells was detected along the path of the migrating nematodes (Fig. 5A). A strong expression of GFP was observed in syncytia at 15 and 24 hai (Fig. 5B, C), whereas non-infected pAP2C1::AP2C1-GFP plants showed a much weaker fluorescence signal throughout the studied root tissue compared with the signal detected in syncytia (Fig. 5D).

In summary, these data show enhanced expression of *AP2C1* and increased AP2C1 amounts during nematode migration and ISC selection.

### Response to *H. schachtii* is modulated in *mpk3*, *mpk6*, and *ap2c1* plants

**Fig. 6.**
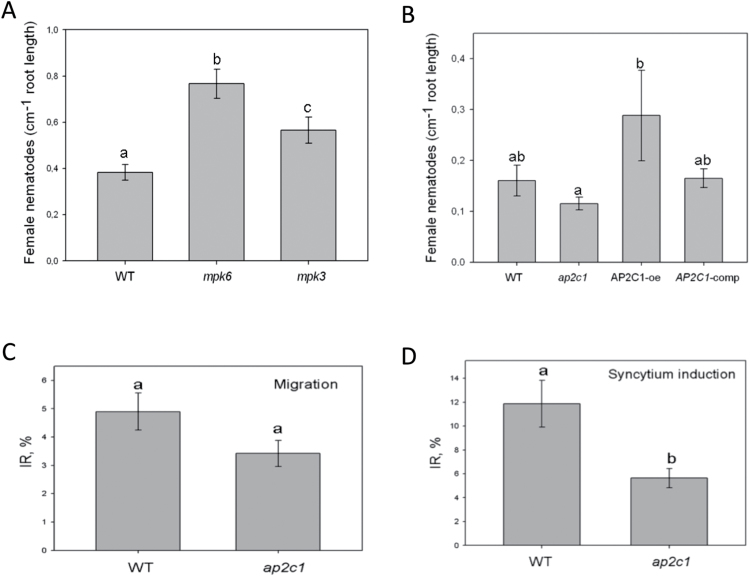
*H. schachtii* infection assays. (A) Number of female nematodes per root centimetre in Col-0, *mpk6* and *mpk3* mutant lines. (B) Number of female nematodes per root centimetre in Col-0, *ap2c1*, AP2C1-oe, and AP2C1-comp lines. (C) Percentage of migrating J2s in Col-0 and a*p2c1* roots (2–3hai). (D) Infection rates in Col-0 and a*p2c1* roots at 6 hai (during syncytium induction). Values are means ±SE based on three replicates. Letters represent significant differences: *P* < 0.05 versus control by ANOVA.

The results presented above show the enhanced activation of MAPKs in the absence of AP2C1 and higher *AP2C1* expression in nematode-infected host roots. Thus, the importance of these MAPK signalling components during the *H. schachtii*–*Arabidopsis* interaction was tested. It was investigated whether the lack of MPK3 and MPK6 affects nematode development in plants. Nematode infection tests were performed on *mpk6* and *mpk3* mutant lines compared with the wild type. Our data show a significant increase in nematode development (number of female nematodes) in both *mpk3* and *mpk6* mutants compared with the wild-type plants (Fig. 6A), suggesting a positive role of both MPK3 and MPK6 in plant defence against the nematodes. To evaluate the role of AP2C1 phosphatase in the development of *H. schachtii*, *ap2c1* and AP2C1-oe lines were tested and compared with the wild type and *ap2c1*-complemented line (AP2C1-comp). Our results show that significantly fewer nematodes were developing in *ap2c1* than in the AP2C1-oe line (Fig. 6B). The development of nematodes was similar in the wild type and the complemented line.

Comparing the percentage of migrating and syncytium-inducing J2s revealed significant differences between the wild-type and *ap2c1* plants. Our data show that significantly fewer nematodes entered the roots of *ap2c1* compared with wild-type plants (2–3 hai, Fig. 6C). Consequently, at 6 hai, significantly more J2s started to induce syncytia in the wild type than in *ap2c1* (Fig. 6D).

**Fig. 7.**
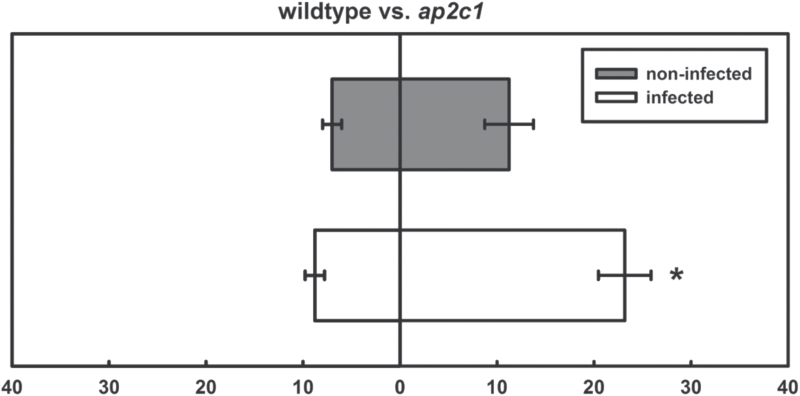
Nematode attraction assay. Comparison of the attractiveness of exudates collected from roots of non-treated or nematode-infected roots of wild type and *ap2c1* plants, respectively. Values are means ±SE (*n*=3). Asterisk represents significant difference: *P* <0.05 (ANOVA).

Since significant differences between infection rates of *ap2c1* and the wild type were observed, nematode attraction assays were performed by comparing the attractiveness of both lines towards migrating J2s (Fig. 6). It was found that exudates of non-treated roots from all lines were similarly attractive to J2s. However, comparing root exudates from both lines attacked by J2s, a clearly enhanced attractiveness of *ap2c1* to nematodes is shown (Fig. 7).

## Discussion

Various DAMPs are released from plant cell walls during mechanical wounding (Newman *et al.*, 2013) and most likely during the attack of plant-parasitic nematodes such substances are generated as a result of enzymatic digestion and stylet thrusting that cause severe damage to the plant cell walls. The role of nematode-related molecules in the induction of plant defence responses is frequently discussed, suggesting that nematode-specific PAMPs could be, for instance, chitin or components of the nematode cuticle (Libault *et al.*, 2007; Lozano and Smant, 2011; Smant and Jones, 2011; Quentin *et al.*, 2013). Both DAMPs and PAMPs induce rapid and specific activation of *Arabidopsis* MAPKs (Galletti *et al.*, 2011; Meng and Zhang, 2013), however, to date, there is no clear evidence of nematode-specific PAMPs or DAMPs and it is not known if nematodes are triggering the activation of plant MAPK signalling. Therefore, the focus here was on the early stages of sugar beet cyst nematode *H. schachtii* root infection and specific wound- and nematode-induced activation of *Arabidopsis* MPK3 and MPK6 has been demonstrated. In addition, the transcriptional induction of the MAPK phosphatase *AP2C1* is shown in nematode-infected tissue suggesting a negative feedback loop for the kinase cascade in roots, similar to observations in leaves (Meskiene *et al.*, 1998; Schweighofer *et al.*, 2007).

Comparison of the MAPK activation mode by nematode and by mechanical wounding indicates both parallels and remarkable specificities.

### Early nematode infection induces specific activation of MAPKs

Our results show very strong and transient MPK3/MPK6 activities in plant roots immediately after puncturing root epidermal cells with the needle as well as MPK6 activity in response to cellulase (β-1,4-endoglucanase). Cellulases are important components of nematode saliva (Smant *et al.*, 1998) and facilitate the degradation of plant cell walls in the root cells along the nematode’s migratory track (Wang *et al.*, 1999). During nematode infection, the first MAPK activation was detected as early as during the entrance of nematodes into the root tissue indicating that the plant perceives the parasite at the onset of the infection. In contrast to the wound- or cellulase-induced MAPK response, which peaks within minutes and then is rapidly reduced, the nematode-specific reaction attains the maximum later. High activation of MAPKs decreases during the syncytium formation. The difference between the degree of MAPK activation caused by nematodes and by wounding or cellulase treatment suggests either that damage caused by the nematodes is so minimal that the plant reaction is very weak at that stage or that the nematodes are able to attenuate plant cell signalling. Interestingly, the nematode-induced MPK3 and MPK6 activation profiles differ, suggesting their specific tasks during cell signalling and the induction of the plant innate immunity mechanisms against *H. schachtii*. The extensive intracellular migration performed by J2s along the root and the existence of many potential effectors produced by nematodes (Rosso *et al.*, 2011) suggests a possible suppressive activity of these parasites on MAPKs activation and related downstream defence reactions in plants.

### MAPK signalling is specifically modulated during nematode attack

Our observations suggest that nematodes may attenuate and postpone MAPK activation during the infection process, which thus leads to delayed plant defence reactions. Similarly, induction of the MAPK phosphatase *AP2C1* is attenuated and extended for hours during nematode attack correlating with the delayed MAPK activities after successful infection. Observed modulations of nematode-induced MAPK signalling could be explained by the activities of the nematodes, which produce and inject different effectors that could target DAMP- or PAMP-induced signalling cascades in plant cells during migration and feeding site induction. These effectors are synthesized in the complex secretory gland cells and secreted through the nematode stylet (Hussey, 1989; Davis *et al.*, 2004) or through the cuticle into the plant cells (Rosso *et al.*, 2011). Beside cellulases that act as effector proteins of cyst nematodes (Smant *et al.*, 1998; Rosso *et al.*, 2011), many other secreted proteins from cyst nematodes have been found in affected plant cells to modulate complex changes in plant gene expression for the benefit of the parasite (Rosso *et al.*, 2011). Similarly, plant bacterial pathogens use a variety of secreted virulence factors to control the biological processes in plant host cells that are delivered into the plant cell to modulate cell physiology by inhibiting plant immunity components, altering plant hormone homeostasis or signalling (Melotto and Kunkel, 2013). For instance, *Arabidopsis* MPK4 is targeted by effector protein AvrB from *Pseudomonas syringae* (Cui *et al.*, 2010), an effector protein HopAI1 targets and irreversibly inactivates MPK3, MPK4, and MPK6, suppressing plant immune responses that would inhibit bacterial propagation (Zhang *et al.*, 2012). It can be speculated that, as shown for *P. syringae* effectors, the nematodes may suppress plant defence with the aid of still unknown effectors or may employ intrinsic plant factors, such as AP2C1 to suppress plant cell signalling and defence.

AP2C1 is a PP2C-type MAPK phosphatase, which dephosphorylates p-Thr within the MAPK activation loop (Meskiene *et al.*, 2003; Schweighofer *et al.*, 2009) and represents part of a negative feedback loop in the regulation of MAPK activity (Meskiene and Hirt, 2000). Induction of *AP2C1* during nematode migration in plants corresponds with its response to mechanical wounding or pathogen attack (Schweighofer *et al.*, 2007). However, in spite of the ability of AP2C1 to inhibit efficiently wound- or cellulase-induced MAPKs in roots, during nematode migration, activation rather than inhibition of MAPKs is observed, whereas inactivation is postponed for hours to the timing of syncytia initiation, suggesting a role of the parasites in this modulation. On the other hand, the quantitatively lower expression of *AP2C1* during the early stages of nematode infection compared with wound-induced transcription indicates different plant reactions to damage induced by mechanical wounding than damage by the parasites. Strongly and locally enhanced *AP2C1* expression and AP2C1 protein accumulation at the site of syncytium induction indicates that a negative regulator of MAPKs is amply induced to ensure suppression of MAPK activation in developing syncytia. The higher abundance of AP2C1 protein also strongly suppressed cellulase-induced MAPK activation in roots in AP2C1-oe lines. In the *ap2c1* line, nematode-induced MAPKs activities are stronger and more sustained compared with WT plants. This suggests that the role of AP2C1 is in keeping the threshold of kinase activities and inactivation of MPK6/3 in the WT plants. These enhanced and prolonged kinase activities in *ap2c1* plant roots may lead to defence responses reflected by enhanced plant resistance against the nematodes. Strong reduction of MAPK activities in *ap2c1* plants at 20 hai indicates a contribution of other MAPK phosphatase(s) to dephosphorylate the kinases. These could potentially be Ser/Thr phosphatases, such as other PP2Cs, as well as PTP/DSP family members (Bartels *et al.*, 2010; Fuchs *et al.*, 2013). Reduction of MAPK activities by AP2C1 ectopic expression restrains plant cell signalling leading to the enhancement of plant sensitivity to nematodes, probably due to attenuated plant defence.

Taken together, our observations suggest that nematodes may enhance *AP2C1* expression during the infection process, which finally leads to lower MAPK activities. It cannot be excluded that nematode’s effector(s) could target components of this signalling pathway or AP2C1 protein directly thus modulating its function as a negative regulator of MAPK activities. Nematodes may also efficiently suppress early MAPK-related defence responses by subtle activation of the negative regulator AP2C1, but cannot use this strategy in the absence of AP2C1 in the knockout plants.

### Altered susceptibility towards nematodes in *mpk3*, *mpk6*, and *ap2c1* plants

Studying the role of AP2C1 during infection and nematode development showed enhanced activation of MPK3 and MPK6 in plants lacking AP2C1. These plants were less infected and caused a reduction in the development of both syncytia and nematodes when compared with the AP2C1-oe line, where the opposite situation was observed. Interestingly, altered AP2C1 levels did not significantly affect nematode penetration and intracellular migration in the roots. However, it was possible to show that, as soon as J2s started to induce syncytia, AP2C1-controlled plant defence was activated. Lack of *AP2C1* expression reduced syncytium induction and, finally, influenced nematode development to some extent. We suggest that the increase in MPK3 and MPK6 activities as observed in *ap2c1* plants leads to improved defence against the nematodes and the lack of these MAPKs or their activities provide more favourable conditions for *H. schachtii*. This is similar to previous reports showing that AP2C1-oe plants exhibit reduced defence responses and are more susceptible to penetration and colonization by *B. cinerea* than wild-type and *ap2c1* plants (Schweighofer *et al.*, 2007; Galletti *et al.*, 2011). Together with enhanced activation of MAPKs, *ap2c1* mutants showed a higher level of wound-induced JA and were more resistant to the phytophagous mite, *Tetranychus urticea* (Schweighofer *et al.*, 2007). These enhanced defence responses exhibited by the mutant may explain the reduced susceptibility to nematodes observed in our work. Plants with elevated JA levels, in general, are more resistant to herbivores and pathogens (Glazebrook, 2005; Liechti *et al.*, 2006), including cyst and root-knot nematodes (Oka *et al.*, 1997; Thurau *et al.*, 2003; Soriano *et al.*, 2004). Cooper *et al.* (2005) showed that the foliar application of JA induces systemic defence against root-knot nematodes in tomato. An important role of JA in defence against root-knot nematodes was demonstrated in rice (Nahar *et al.*, 2011) and more recently in *Arabidopsis* where JA triggers early defence responses against *H. schachtii* (Kammerhofer *et al.*, 2015).

Nematode-induced plant hormone synthesis and other plant defence responses may also attract other nematodes. Indeed, it was shown recently that J2s of *H. schachtii* are more attracted towards plants that are already infected with nematodes than non-infected controls. Moreover, the authors showed that, at the early stages of nematode parasitism, the levels of JA and ET are significantly elevated (Kammerhofer *et al.*, 2015). Similarly, root exudates of plants showing increased ET production were more attractive to *H. schachtii* infective J2s (Wubben *et al.*, 2001). Thus, nematodes may use ET to communicate the successful infection of susceptible hosts to other individuals. *ap2c1* mutant did not produce significantly enhanced levels of ET in leaves after wounding, however, it may produce more ET after nematode infection. Currently, it is not known which chemicals attract nematodes, but water-soluble and volatile compounds are being suggested (Ali *et al.*, 2011; Farnier *et al.*, 2012). Since nematodes are more attracted by previously infected roots, it is likely that compounds produced during an ongoing nematode attack may enhance root attractiveness towards following J2s. Accordingly, the root exudates of the nematode-infected *ap2c1* line, showing higher MAPK activities, are more attractive to following J2s than exudates of infected wild-type roots. These results suggest that silencing *AP2C1* expression may present a promising strategy to produce nematode trap crops, which attract nematodes but hamper their further development.

In conclusion, this study presents new evidences of plant–nematode perception and the following activation of MAPK signalling pathways in *Arabidopsis*. It shows a negative role of the AP2C1-regulated MPK3 and MPK6 in nematode development and a role of the protein phosphatase AP2C1 during the early stages of *H. schachtii* parasitism. Considering prospective discoveries of nematode PAMPs and new effectors, as well as nematode-specific receptors acting upstream of the MAPK cascade, plant cell signalling studies are not only opening new and exciting possibilities to study plant–nematode interactions, but may also reveal some plant-specific protection mechanisms against these parasites. Further detailed investigations of plant cell signalling mutants with the focus on nematode root migration might shed more light on how the host perceives nematodes.

## Supplementary data

Supplementary data can be found at *JXB* online.

Supplementary Fig. S1. Analysis of MAPKs activation in roots during mechanical wounding.

Supplementary Fig. S2. Analysis of MPK3 and MPK6 activation in mechanically wounded roots of Col-0, *mpk3*, and *mpk6* mutant lines.

Supplementary Fig. S3. Quantification of MPK3 and MPK6 activation during early stages of *H. schachtii* infection.

Supplementary Data
