# Supplementary material for: Plant resistance against the parasitic nematode Heterodera schachtii is mediated by MPK3 and MPK6 kinases, which are controlled by the MAPK phosphatase AP2C1 in Arabidopsis
Source: J Exp Bot. 2015 Oct 5;67(1):107–18. doi: 10.1093/jxb/erv440 (PMC4682428; doi:10.1093/jxb/erv440)
Supplement: Supplementary Data [file supp_67_1_107__index.html]

Plant resistance against the parasitic nematode Heterodera schachtii is mediated by MPK3 and MPK6 kinases, which are controlled by the MAPK phosphatase AP2C1 in Arabidopsis — Plant resistance against the parasitic nematode Heterodera schachtii is mediated by MPK3 and MPK6 kinases, which are controlled by the MAPK phosphatase AP2C1 in Arabidopsis — Supplementary Data 

# Plant resistance against the parasitic nematode *Heterodera schachtii* is mediated by MPK3 and MPK6 kinases, which are controlled by the MAPK phosphatase AP2C1 in Arabidopsis

## Supplementary Data

Data files

- Supplementary Data - Supplementary Data
