# Supplementary material for: Plant resistance against the parasitic nematode Heterodera schachtii is mediated by MPK3 and MPK6 kinases, which are controlled by the MAPK phosphatase AP2C1 in Arabidopsis
Source: J Exp Bot. 2015 Oct 5;67(1):107–18. doi: 10.1093/jxb/erv440 (PMC4682428; doi:10.1093/jxb/erv440)
Supplement: Supplementary Data [file supp_erv440_jexbot150797_file001.pdf]

Plant resistance against the parasitic nematode *Heterodera schachtii* is mediated by MPK3 and MPK6 kinases, which are controlled by the MAPK phosphatase AP2C1 in Arabidopsis

Ekaterina Sidonskaya, Alois Schweighofer, Volodymyr Shubshynskyy, Nina Kammerhofer, Julia Hofmann, Krzysztof Wieczorek, Irute Meskiene

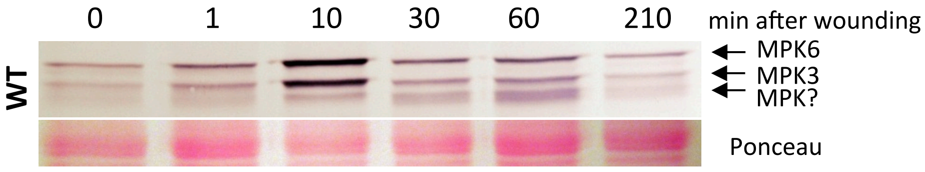

Supplementary Figure S1. Analysis of MAPKs activation in roots during mechanical wounding.
Phosphorylation of MPK6 and MPK3 was detected by immunoblotting with anti-phospho ERK1/2 antibody. Ponceau-stained membranes present protein loading.

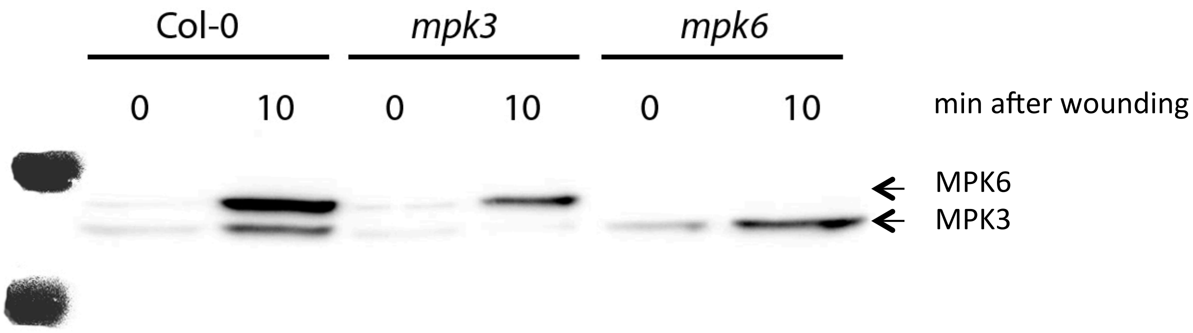

Supplementary Figure S2. Analysis of MPK3 and MPK6 activation in mechanically wounded roots of Col-0, *mpk3* and *mpk6* mutant lines. Phosphorylation of MPKs was detected by immunoblotting with anti-phospho ERK1/2 antibody.

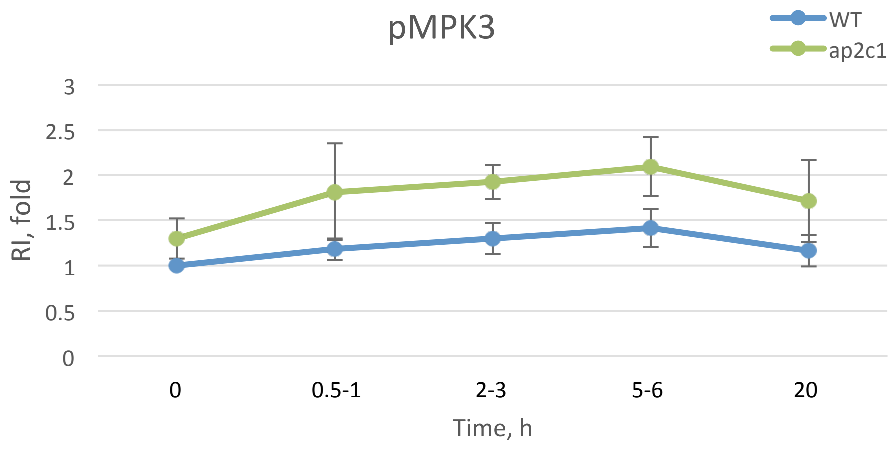

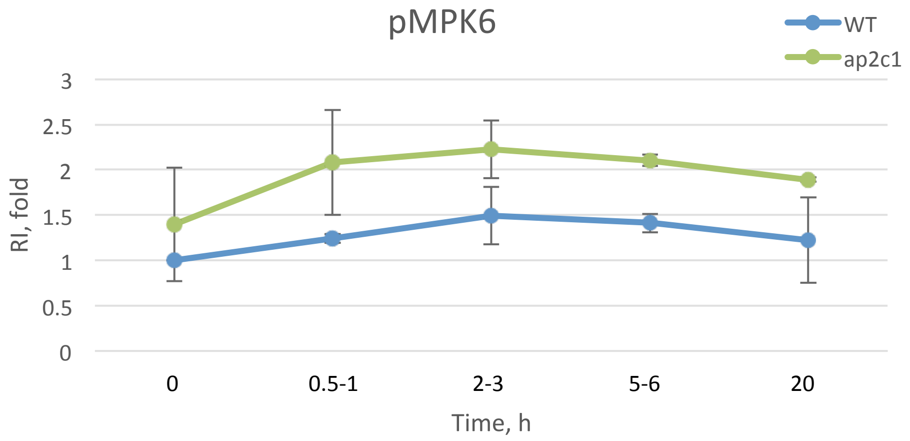

Supplementary Figure S3. Quantification of MPK3 and MPK6 activation during early stages of *H. schachtii* infection**.** Values shown are means +/- standard deviation from three independent repetitions. The quantification was performed with ImageJ, RI – relative intensity.
